# Glyceroniosomes for enhanced intestinal absorption of hydrochlorothiazide and lisinopril in their fixed dose combination

**DOI:** 10.1038/s41598-024-74986-1

**Published:** 2024-10-18

**Authors:** Aya R. Elbasuony, Abdelaziz E. Abdelaziz, Eman A. Mazyed, Gamal M. El Maghraby

**Affiliations:** 1 Department of Pharmaceutical Technology, Faculty of Pharmacy, Kafrelsheikh University, Kafrelsheikh, Egypt; 2 Faculty of Pharmacy, Tanta University, Tanta, Egypt

**Keywords:** Fixed dose combination, Hydrochlorothiazide, Lisinopril, Glyceroniosome, In situ intestinal perfusion, Pharmaceutics, Drug delivery

## Abstract

The objective was to investigate the effect of co-administration of hydrochlorothiazide and lisinopril as fixed dose combination on their intestinal absorption. The scope was extended to enhance intestinal absorption of both drugs. In situ rabbit intestinal absorption through the duodenum and jejuno-ileum was used to monitor membrane permeability of both drugs when perfused alone or in combination. Niosomes containing glycerols (glyceroniosomes) were loaded with both drugs. Glyceroniosomes comprised Span 60 or Tween 40 in combination with cholesterol and glycerol were prepared by bath sonication. Glyceroniosomes were characterized with respect to vesicle size, drug entrapment efficiency and were examined using transmission electron microscope (TEM). The prepared vesicles were nanosized spherical vesicles with average size of 202.4 nm and 108.8 nm for span free and span containing glyceroniosomes, respectively. The recorded Zeta potential values suggested good stability of the prepared formulations. Intestinal absorption studies reflected incomplete absorption of hydrocholothiazide and lisinopril correlating with their categorization as class IV and III drugs, respectively. Co-perfusion of both drugs reduced the intestinal absorption of lisinopril. Simultaneous encapsulation in glyceroniosomes enhanced the intestinal absorption of both drugs. Tween based systems were more efficient. The study introduced glyceroniosomes as carriers of simultaneous delivery of hydrochlorothiazide and lisinopril.

## Introduction

Treatment of chronic diseases requires administration of more than one active ingredient which can be formulated in separate dosage forms. This can negatively influence patients’ compliance to treatment protocol. Fixed dose combinations (FDC) which involves production of more than therapeutically related drug in the same dosage form was adopted to solve this problem^1,2^. FDC is widely used due to recorded efficacy and patient compliance and reduced cost. The success was specifically shown in treatment of hypertension and other cardiovascular diseases in addition to multivitamins^3,4^. Research studies indicated possible achievement of suboptimal effectiveness and unexpected side effects and safety issues^5^. The reasons for unexpected efficacy and side effects require investigation.

Hydrochlorothiazide is one of the most widely used diuretics in FDC. It is available in combination with lisinopril which is orally active angiotensin‐converting enzyme inhibitor (ACEI). This combination is employed for management of hypertension^6,7^. Both drugs in this combination are poorly bioavailable after oral administration. Hydrochlorothiazide has poor permeability with low solubility and thus included in class IV category according to biopharmaceutical classification system (BCS)^8^. These features resulted in its variable and low oral bioavailability^9,10^. Lisinopril is a hydrophilic with poor membrane permeability that belongs to class III according to BCS. This contributed to its low oral bioavailability which is about 25%^6,11–13^. Moreover, the effect of simultaneous administration of both drugs on their intestinal absorption requires investigation. Accordingly, the first aim of this study was to probe the effect of simultaneous delivery of hydrochlorothiazide and lisinopril on their intestinal absorption. This employed in situ rabbit intestinal perfusion technique which allows investigation of membrane transport parameters and the mechanisms of drug absorption. Reasons for poor absorption can be obtained from this technique^14,15^. The work was extended to enhance intestinal absorption of both drugs.

Nanotechnology opened the gate for enhanced oral bioavailability of poorly bioavailable drugs. Promising nanocarriers include solid lipid nanoparticles, microemulsions and vesicular systems which include liposomes and niosomes^16–19^. Vesicular systems have the advantage of being able to carry both hydrophilic and lipophilic drugs simultaneously. Liposomes were initially developed and were widely tested in various routes of administration including oral route^20,21^. Glycerol was included in liposome components to produce the so called glycerosomes. These glycerosomes were shown to improve the biopharmaceutical properties of liposomes with high potential for hastened membrane permeability but this was not tested orally^22,23^. Niosomes are cheaper and more stable substitutes for liposomes. They have been shown to enhance the intestinal absorption of drugs^24,25^. Inclusion of glycerol into their composition may enhance its efficacy as shown in liposomes but this requires verification. Thus the current study tested glyceroniosomes as vesicular carriers for enhanced intestinal absorption of hydrochlorothiazide and lisinopril. This study is the first to prepare and characterize glyceroniosomes.

## Materials and methods

### Materials

Hydrochlorothiazide and Lisinopril were obtained as gift from South Egypt Drug Industries Co (SEDICO). Methanol and ethanol (HPLC – grade) were procured from Sigma Aldrich Chemical Co., Missouri, USA. Potassium dihydrogen phosphate, potassium chloride, disodium hydrogen phosphate and sodium chloride were obtained from El-Nasr Pharmaceutical Chemicals Company, Cairo, Egypt. Sorbitan monostearate (Span 60) was obtained from Oxford Lab Chemicals, Mumbai, India. Cholesterol was acquired from Advent Chembio Pvt. Ltd., Mumbai, India. Polyoxyethylene Sorbitan Monopalmitate (Tween 40) was also gained from Qualikems Fine Chem Pvt. Ltd., Nandesari, Vadodara, India. Ketamine hydrochloride injection was attained from Rotex Medica., Trittau, Germany.

### Methods

#### High pressure liquid chromatography (HPLC)

Hydrochlorothiazide and lisinopril were simultaneously quantified using in house developed method. This employed A Dionex UltiMate 3000 HPLC (Thermo Scientific™, Dionex™, Sunnyvale, CA, USA). The instrument employs a quaternary pump (LPG-3400SD), an auto sampler (WPS-3000TSL), a column thermostat (TCC- 3000SD), and a diode array detector (DAD). The samples (30 µl) were injected into the chromatogram passing through ODS column 15 cm length, 0.46 cm internal diameter and an average particle size of 5 µm as stationary phase (GL Sciences Inc., Japan). This column was maintained at 35 °C. The mobile phase constituted 0.02 M of potassium dihydrogen phosphate buffer (adjusted to pH 7.5 with potassium hydroxide solution) and methanol (80:20). This was introduced at a rate of 1 ml/minute and the concentration of drug(s) was quantified at 210 nm. The process is fully computerized using Chromeleon 7 software.

The method was validated for linearity, selectivity, precision and lower limit of quantification (LOQ).

#### Linearity

Linearity was monitored from the correlation coefficient of the straight line fitted to the peak area versus concentration plots.

#### Accuracy

The closeness of the recovered concentration to the exact value was used as a reflection for the accuracy of the assay. The recovered concentration was expressed as percentage of the expected concentration and acceptable results must be in the range of 98–100%.

#### Precision

The precision is indicated from the Relative Standard Deviation (RSD) which is computed by dividing the SD by the average and expressing it as percentage.

#### Lower limit of detection (LOD)

This can be estimated from the calibration graphs using the following equation^26^:

$${\text{LOD }} = { 3}.{3 } \times {\text{ SD}}_{{{\text{intercept}}}} /{\text{slope}}{.}$$

#### Lower limit of quantitation (LOQ)

This was determined from the calibration curves using the following equation^26^:

$${\text{LOQ }} = { 1}0 \, \times {\text{ SD}}_{{{\text{intercept}}}} /{\text{slope}}{.}$$

#### Preparation of glyceroniosomes

Table 1 presents the composition of the tested glyceroniosome formulations. Glyceroniosomes were fabricated using the documented procedures^27^. Tween 40, Span 60, cholesterol, and the hydrophobic drug (hydrochlorothiazide) were liquefied by heating on water bath to (65 ± 1 °C). Ethanol was added to this system while heating to form clear dispersion. Water (equivalent amount to ethanol) was added with mixing to prepare homogenous dispersion. Lisinopril was solubilized in 10 ml of pre-filtered distilled water before gradual addition to the lipid dispersion. Glycerol was then added with mixing before removing from the water bath and addition of the rest of water to the target volume. This provides crude glyceroniosomal dispersion which was left at ambient temperature for an overnight to undergo complete swelling. The glyceroniosomal dispersion was bath sonicated for 30 min for vesicle size reduction.

**Table 1:** The compositions of the tested formulations expressed as weight ratios.

Formulation	Span free glyceroniosome	Span containing glyceroniosome
Tween 40	2.4	1.8
Span 60	–	0.6
Cholesterol	0.6	0.6
Ethanol	3	3
Glycerol	10	10
Hydrochlorothiazide	0.030	0.030
Lisinopril	0.024	0.024
Water ad	60	60

#### Particle size and zeta potential measurements

The size and zeta potential of prepared vesicles were measured by Zetasizer (Malvern Instruments Ltd, Worcestershire, UK) which depends on dynamic light scattering. Glyceroniosomes were diluted 1 in 100 with distilled water previously filtered through 0.2 µm membrane filter. The instrument was maintained at 25 °C with a diffraction angle of 90°. Size measurement was conducted three times with each run constituting 14 measurement cycles. The collected data were presented as Z-average values (average size) and the particle size distribution was assessed from polydispersity index (PDI). Zeta potential was recorded as a mean of 100 determinations for each sample.

#### Transmission electron microscopy (TEM)

TEM was adopted to research the morphology and to further assess the size of vesicles. This process used TEM obtained from JEOL (JEOL – JSM- 1400 Plus, Tokyo, Japan). The vesicles were diluted with filtered water before loading on copper grids and successive staining with uranyl acetate and lead citrate. The stained sample was examined using TEM and the fields were micrographed^24^.

#### Determination of entrapment efficiency (EE%)

The entrapment efficiency values of hydrochlorothiazide and lisinopril in glyceroniosomes were determined after separation of free compound by dialysis. The vesicular dispersion (2 ml) was loaded in dialysis sacs molecular weight cut off 12-14KDa (Cellulose dialysis tubing, Serva, Germany). Dialysis was conducted by incubation in 50 ml of PBS (pH 7.4) for 4 h at the end of which the concentration of drugs in the dialysate was quantified by HPLC. The amount of drug separated was taken as a measure for the unencapsulated drug. EE% was computed using the following relation in which Ct is the total amount of drug present in the vesicles in the sac, Cf is the amount of free drug^28^:

$${\text{EE }}\% \, = \, \left[ {\left( {{\text{C}}_{{\text{t}}} - {\text{ C}}_{{\text{f}}} } \right)/{\text{C}}_{{\text{t}}} } \right)] \, \times { 1}00$$

#### Preparation of perfusion fluid

The perfusion fluid contained hydrochlorothiazide, lisinopril or their combination. The drugs were prepared as true aqueous solution in PBS or encapsulated in glyceroniosomes. The concentration of drugs in the perfusion solution was kept at 12.5 and 10 µg/ml for hydrochlorothiazide and lisinopril respectively. To prepare glyceroniosomal perfusion solution the crude glyceroniosomal dispersion was bath sonicated for 30 min before dilution with PBS to obtain the required concentrations of hydrochlorothiazide and lisinopril. The pH of the perfusion fluid was adjusted to 6.6 for infusion into duodenum and 7.4 for jejuno-ileum. These fluids were kept at physiologic body temperature (37 °C) which was maintained throughout the experiment.

#### In situ intestinal absorption studies

This study is reported in accordance with ARRIVE guidelines. All methods were performed in accordance with the relevant guidelines and regulations. The study protocol and manipulation of rabbits were approved by the ethical committee, College of Pharmacy, University of Kafrelsheikh (approval number, KFS-IACUC/103/2023). The study utilized fifteen male albino rabbits weighing 2–2.5 kg. The rabbits were purchased from local animal house. The intestinal absorption of drugs was observed from the duodenum and jejuno-ileum. The surgery and preparation of duodenum and jejuno-ileum for in situ perfusion was achieved based on valid procedures^29,30^. This required overnight fasting of rabbit before the surgery. General anesthesia was achieved by IM injection of 20 mg/kg xylazine followed by ketamine HCl (2 successive doses of 45 mg/kg at 15 min intervals). Extra dose of 25 mg/kg ketamine was injected when needed. The rabbit was then rested in a flat position on a heating pad and the abdominal hair was cleaned before longitudinal incision through abdominal midline. The duodenum and the jejuno-ileum were exposed. Duodenum was proximally tied off using surgical thread before measuring 20 cm towards the distal part at which the duodenum was ligated. A three-way stopcock cannula was fitted proximally through and incision with L-shaped glass cannula being mounted distally. The lumen was cleaned using warm saline. The jejuno-ileum segment (30 cm) was prepared using similar procedures. Both segments were employed simultaneously in each rabbit. To guarantee steady flow of perfusate the intestinal segment was carefully ordered in a uniform S- to multi-S pattern. Body temperature and hydration were maintained by covering the exposed area with gauze pad that was wetted periodically with saline previously adjusted to 37 °C. The perfusion liquid (drug solution or its glyceroniosomal formulation) was pumped at constant flow rate (0.27 ml/ min using Harvard-22; Harvard Apparatus, Millis, Massachusetts, USA). The effluent was collected every 10 min for 2 h, and volume of the sample was precisely measured. The animal was sacrificed with sharp surgical scalpel under anesthesia at the end of the experiment and the actual length of each segment was measured accurately. The collected samples were centrifuged to precipitate any mucus debris. The supernatant (30 µl) was injected into the HPLC to measure the drug(s) concentration.

### Data analysis

#### Absorptive clearance

Detailed description of in situ intestinal perfusion data analysis is available elsewhere in previous publications^15,28^. The pump flow rate was adopted to compute the expected sample volume. This volume was to compute water flux by subtracting the real volume of perfusate from it. The calculated water flux was adopted to normalize the recorded drug concentration for volume change to accurately compute the real amount of drug remaining per unit time {C(out)}. The amount of drug perfused per minute {C(in)} is computed by multiplying the initial drug concentration in the perfusion fluid by perfusion rate. The ratio between {C(out)}and {C(in)} was computed for the samples collected in the second hour to provide the steady state fraction remaining after perfusion [{C(out)/C(in)}ss]. The fraction remaining after perfusion was used to determine the permeability-surface area product (PeA) according to Eqs. 1 and 2^31,32^:

1 $$\left\{ {{\text{C}}\left( {{\text{out}}} \right)/{\text{C}}\left( {{\text{in}}} \right)} \right\}{\text{ss}} = {\text{ exp}}^{{ - \, ({\text{PeA}}/{\text{Q}})}}$$

2 $${\text{PeA }} = \, - {\text{ Q }}*{\text{ ln}}\left\{ {{\text{C}}\left( {{\text{out}}} \right)/{\text{C}}\left( {{\text{in}}} \right)} \right\}{\text{ss}}$$

where Pe is the apparent permeability coefficient (cm/min), A is the effective surface area (cm^2^) and Q is the mean trans-intestinal flow rate (ml/min).

The fraction absorbed (Fa) was determined according to Eq. 3.

3 $${\text{Fa }} = { 1 } - \left\{ {{\text{C}}\left( {{\text{out}}} \right)/{\text{C}}\left( {{\text{in}}} \right)} \right\}{\text{ss}} = { 1 } - {\text{ exp}}^{{ - ({\text{PeA}}/{\text{Q}})}}$$

Anatomical reserve length (ARL) which is intestinal length remaining after complete drug absorption is estimated using Eq. 4 to reflect the magnitude of drug absorption with positive value of ARL indicating complete drug absorption from given segment. The maximum intestinal length (cm) available for absorption (L*) and the length of the intestine required for complete drug absorption (l*) were used in this equation. Employing logarithmic equations, drug concentration remaining in the lumen cannot reach zero at the intestinal length (l*). Therefore, l* was approximately estimated from Eq. 5 which was deduced from Eq. 1, assuming 5% fraction of drug remaining as representative for complete drug absorption.

4 $${\text{ARL }} = {\text{ L}}* - {\text{ l}}*$$

5 $$0.0{5 } = {\text{ exp}}^{{ - ({\text{PeA}}.{\text{l}}*/{\text{Q}})}}$$

where PeA is the same as in Eq. 1 but normalized to length, and l* is the length required for 95% absorption (L95%) of the drug.

#### Effect of water flux on intestinal absorption

The overall drug absorption can be via transcellular and/or paracellular pathways with paracellular being directly related to water flux effect. Thus, drug absorption rate “Js” can be expressed using Eq. 6 which studies both the transcellular (diffusive transport) “Ks(C -Cp)” and the paracellular (convective passage) “ФsJwC”^30^.

6 $${\text{Js }} = {\text{ Ks}}\left( {{\text{C }} - {\text{Cp}}} \right) \, + \, \Phi {\text{sJwC}}$$

where “Ks” is the diffusive permeability coefficient, “C” is concentration of the drug in the intestinal lumen while “Cp” is the drug concentration in the plasma. “Фs” is the drug sieving coefficient and “Jw” is the water flux. This equation can be simplified to relation 7 based on the sink conditions of the blood at steady state, where “Jss” is the drug absorption rate at steady state (mg/min) and “Css” is the remaining drug concentration at steady state (mg/ml).

7 $${\text{Jss }} = {\text{ KsCss }} + \, \Phi {\text{sJwCss}}$$

Rearrangement of Eq. 7 gives the Eq. 8 in which the overall absorptive clearance of drug is represented by the term Jss/Css which is determined experimentally as PeA (see above).

8 $${\text{Jss}}/{\text{Css }} = {\text{ Ks }} + \, \Phi {\text{sJw}}$$

Accordingly, plotting of PeA as a function of the Jw will provide a straight line slope of which accounts the sieving coefficient of drug and its Y intercept estimates the diffusive part of the absorptive clearance which provide the contribution of transcellular pathway in drug absorption^33^. The relative contribution of paracellular pathway can be determined by subtracting the Y intercept from the overall absorptive clearance and expressing the product as percentage of the overall PeA.

## Results and discussion

### Chromatography

The developed HPLC was able to simultaneously quantify both hydrochlorothiazide and lisinopril by simple isocratic elution. Hydrochlorothiazide was separated as after 4.1 min as sharp symmetric peak while lisinopril was separated after 6.5 min. The method was linear for both drugs in the concentration range of 1–20 µg/ml. The regression equation for the constructed calibration curve of hydrochlorothiazide was Y = 1.695X − 0.8809. This regression equation was Y = 0.8637X − 0.2759 for lisinopril. The accuracy of the assay for hydrochlorothiazide was reflected from the % drug recovery which was in the range of 99% -103.1% (intraday) and was in the range of 97.8–104% (interday). The precision was shown from the RSD values which ranged from 0.2 to 3.9% (intraday) and from 0.2% to 2.3% (interday). The LOD and LOQ were 0.145 and 0.439 respectively. For lisinopril the accuracy was indicated from the % drug recovery which was in the range of 98.6–103% (intraday) and was in the range of 98.2–102.5% (interday). The precision was shown from the RSD values which ranged from 0.4 to 2.9% (intraday) and from 0.3 to 1.7 (interday). The LOD and LOQ were 0.091 and 0.275, respectively.

### Particle size and zeta potential measurements

**Fig. 1:**
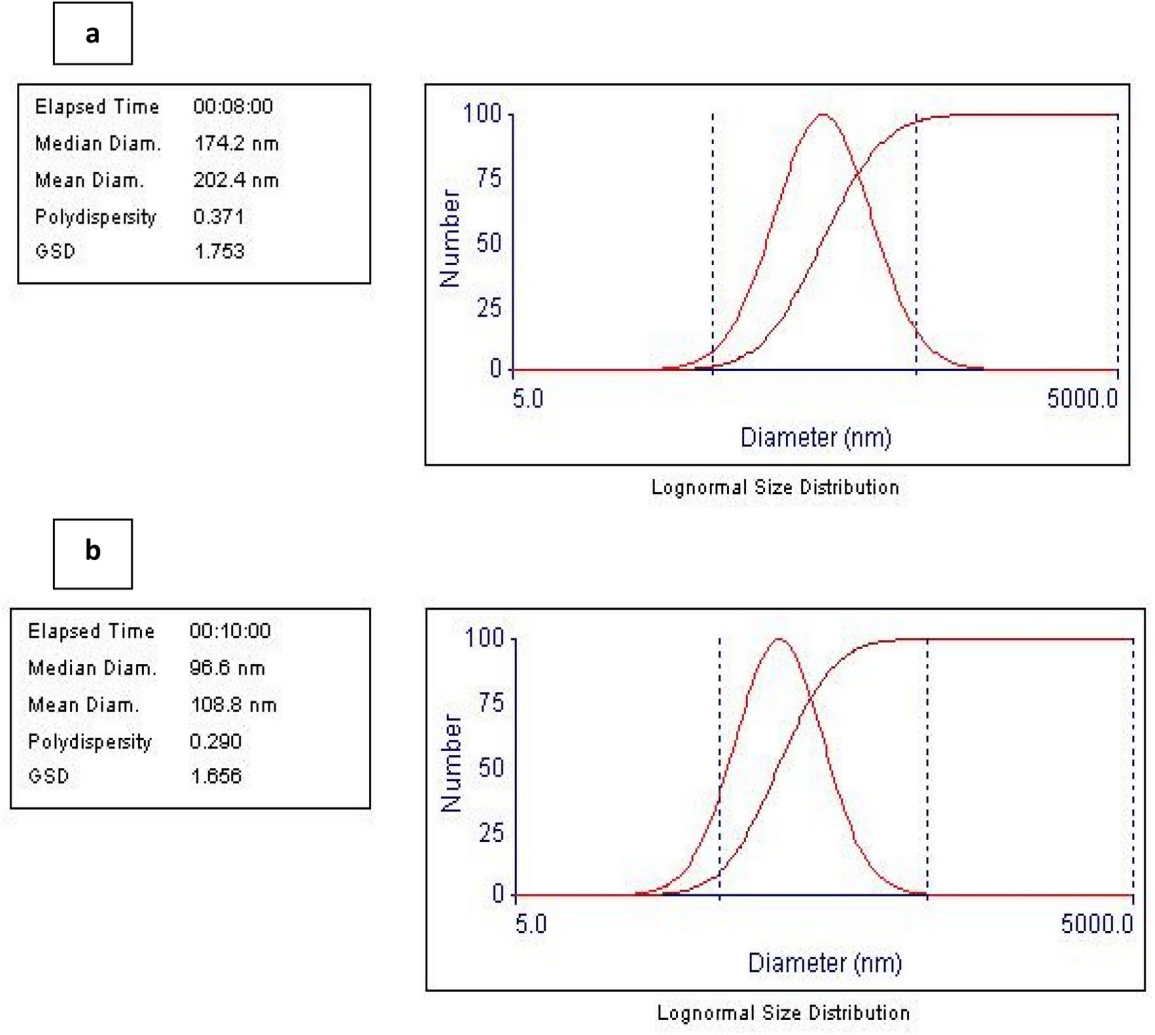
Particle size distribution of span free glyceroniosome (**A**) and span containing glyceroniosome (**B**). Formulation details are in Table 1.

Figure 1 shows the particle size distribution of the tested glyceroniosomes. The mean vesicular size of the prepared glyceroniosomes was recorded to be 202.4 nm ± 1.8 and 108.8 ± 1.7 nm for span free glyceroniosomes and span containing glyceroniosomes, respectively. The vesicles were characterized as heterogeneous population as indicated from PDI values which were 0.37 and 0.29 for span free glyceroniosomes and span containing glyceroniosomes, respectively. The heterogamous distribution is acceptable taking into consideration the technique of preparation which involved hydration of proniosomes followed by bath sonication. It is well established that hydration of vesicles provides crude vesicular systems the size of which is reduced by bath sonication. However bath sonication is known to reduce vesicle size but provide heterogeneous population as shown in the current study. Similar particle size distribution has been shown by other investigators who adopted the same technique^34^. The recorded lower size of span containing vesicles can be attributed to the rigid structure of span 60 which can reduce the degree of vesicles swelling. Presence of Tween 40 instead of Span 60 imparts flexibility to vesicular membrance providing greater chance for vesicle swelling which explains the recorded relatively higher particle size of Span free glyceroniosomes. The recorded Zeta potential values were − 31.8 mV ± 8.8 and − 29.5 mV ± 6.5 for span free glyceroniosomes and span containing glyceroniosomes, respectively. These values suggest good stability of the prepared formulations. The recorded negative zeta potential is expected taking into consideration the composition of which contains components having hydroxyl groups which carries δ–ve charge^35^.

### Transmission electron microscopy (TEM)

**Fig. 2:**
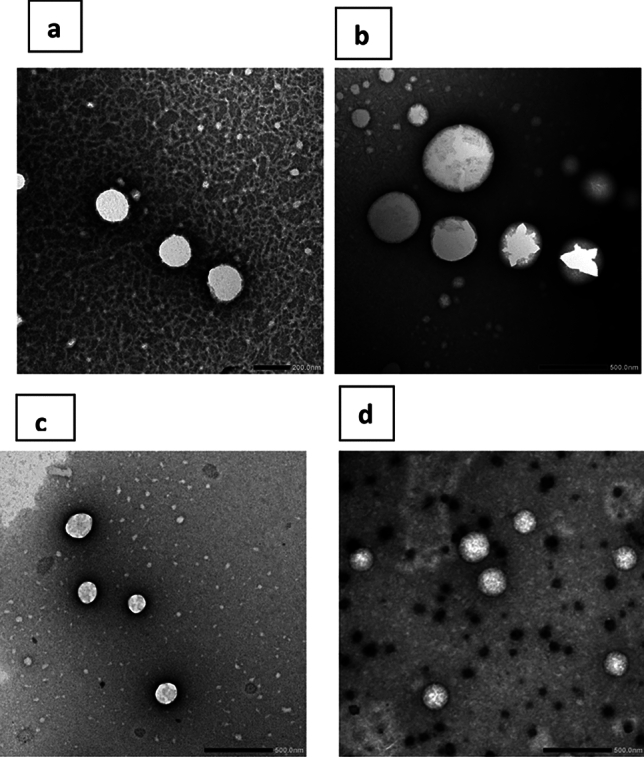
Transmission electron micrographs of span free glyceroniosome (**A**–**B**) and span containing glyceroniosome (**C**–**D**). Formulation details are in Table 1.

Figure 2 shows representative transmission electron micrographs of span free and span containing glyceroniosomes. These micrographs showed glyceroniosomes as spherical nanostructures free from any aggregates. This morphology was preserved irrespective to the composition of the surfactant vesicles. The mean particle size values for the captured nanostructures were calculated to be 210.1 ± 113.2 nm and 184 ± 24.2 nm for Tween 40 glyceroniosomes and Span 60/Tween 40 system. The size of vesicles showed the same rank as recorded by photon correlation spectroscopy but Span/Tween based system was of higher size. The discrepancy can be related to the fact that zetasizer measures the average size of large population of particles but TEM monitor the size of captured fields. Similar pattern has been reported in other research articles^36,37^.

### Determination of entrapment efficiency (EE%)

EE% determination was achieved by isolation of the unentrapped drugs and the results were presented as percentage of drug added as in Table 2. The EE% values of the tested drugs in span free glyceroniosomes were 46.5% and 27.7% for hydrochlorothiazide and lisinopril respectively. Encapsulation of these drugs in span containing glyceroniosomes showed entrapment efficiency values of 40.3% and 27.1% for hydrochlorothiazide and lisinopril respectively. The recorded entrapment efficiency values can be explained on the base of the physicochemical properties of the drug with the hydrophilic drug lisinopril which is expected to locate in the aqueous core of the vesicles showing lower entrapment efficiency compared with that of the more lipophilic hydrochlorothiazide. Noteworthy, the recorded EE% of hydrochlorothiazide is lower than expected for such lipophilic drug which is expected to be preferentially intercalated within the lipid bilayer. The discrepancy can be attributed to the presence of some of the Tween 40 as free monomers in the continuous aqueous phase which can subsequently increase the existence of hydrochlorothiazide in this aqueous phase. This may reduce the entrapment efficiency. Another possible reason for the unexpected reduction of the entrapment efficiency of the lipophilic hydrochlorothiazide is the presence of glycerol which can increase the solubility of drug in the aqueous phase. The dependence of preferential distribution of drugs in vesicular systems on the physicochemical properties of the drug has been postulated after investigation of drug location in liposomes. The authors utilized the polar surface area (PSA) as indicator with polar compound (high PSA) existing in the aqueous core^38^. High entrapment efficiency was shown by other investigators for lipophilic drugs with the hydrophilic ones experiencing low entrapment efficiency^39,40^.

**Table 2:** The entrapment efficiency (EE%) of hydrochlorothiazide and lisinopril from span free glyceroniosome and span containing glyceroniosome.

Entrapment efficiency (EE%)	Span free glyceroniosome	Span containing glyceroniosome
Hydrochlorothiazide	46.5 (0.81)	40.3 (1.28)
Lisinopril	27.7 (1.33)	27.1 (1.72)

Values between brackets are SD (n = 3).

### In situ intestinal absorption studies

The aim of this study was to probe glyceroniosomes for enhanced simultaneous absorption of hydrochlorothiazide and lisinopril through rabbit intestine. The absorption of hydrochlorothiazide and lisnopril was monitored from aqueous solution through the duodenum and jejuno-ileum segments. This was conducted for each drug alone and in combination. Simultaneous absorption was also probed after encapsulation into glyceroniosomes. Rabbit was utilized as model animal based on physiological similarity with human. The technique offers many advantages including elimination of stomach related factors while maintaining blood flow and tissue viability^14,30^. The recorded permeation parameters through the duodenum and jejuno-ileum are presented in Tables 3 and 4.

**Table 3:** Membrane transport parameters of hydrochlorothiazide in the perfused intestinal segment.

	Control		Fixed dose combination	
Parameter	Duodenum	Jejuno-ileum	Duodenum	Jejuno-ileum
PeA/L (mL/min.cm)	0.0018 (0.00048)	0.0011 (5.2145 × 10^–6^)	0.0011 (0.00026)	0.00072 (4.4485 × 10^–5^)
R out/Rin	0.8760 (0.034)	0.8802 (0.0018)	0.9245 (0.0192)	0.9229 (0.0073)
L95% (cm)	784 (160)	1426 (252)	904 (224)	1531 (216)
% Fa/L (%cm^−1^)	0.6528 (0.1668)	0.4084 (0.0045)	0.4069 (0.097)	0.2703 (0.0190)
JW/L (mL/min.cm)	0.0014 (0.00028)	0.001 (0.00028)	0.0004 (0.00035)	0.0008 (0.00021)
ARL (cm)	− 764	− 1246	− 884	− 1351

	Span free glyceroniosome		Span containing glyceroniosome	
Parameter	Duodenum	Jejuno-ileum	Duodenum	Jejuno-ileum
PeA/L (mL/min.cm)	0.0064 (0.00063)	0.0038 (0.00057)	0.0034 (0.00044)	0.0025 (0.000098)
R out/Rin	0.6511 (0.0274)	0.6711 (0.054)	0.7844 (0.0253)	0.7622 (0.0059)
L95% (cm)	136 (16)	228 (34)	266 (37)	333 (7)
% Fa/L (%cm^−1^ )	1.8425 (0.167)	1.1536 (0.148)	1.0589 (0.1194)	0.8199 (0.0136)
JW/L (mL/min.cm)	− 5 × 10^−4^ (0.00039)	0.0003 (0.000121)	0.0001 (0.00025)	0.0006 (0.00025)
ARL (cm)	− 116	− 48	− 246	− 153

Values between brackets represent standard error to the mean (SE) (n = 3).

**Table 4:** Membrane transport parameters of lisinopril in the perfused intestinal segment.

	Control		Fixed dose combination	
Parameter	Duodenum	Jejuno-ileum	Duodenum	Jejuno-ileum
PeA/L (mL/min.cm)	0.0008 (0.000057)	0.0012 (0.00011)	0.0008 (0.000075)	0.00049 (0.000121)
R out/Rin	0.9483 (0.0049)	0.866 (0.01235)	0.9462 (0.0071)	0.9465 (0.01447)
L95% (cm)	1492 (200)	780 (80)	1269 (51)	3189 (742)
% Fa/L (%cm^−1^)	0.2766 (0.0218)	0.4538 (0.03967)	0.2895 (0.02933)	0.1871 (0.0467)
JW/L (mL/min.cm)	0.0006 (0.00016)	0.001 (0.000184)	0.0006 (0.00032)	0.0008 (0.00021)
ARL (cm)	− 1472	− 600	− 1249	− 3009

	Span free glyceroniosome		Span containing glyceroniosome	
Parameter	Duodenum	Jejuno-ileum	Duodenum	Jejuno-ileum
PeA/L (mL/min.cm)	0.0048 (0.000744)	0.0028 (0.00027)	0.0042 (0.00049)	0.0041 (0.00014)
R out/Rin	0.7246 (0.0305)	0.75 (0.01122)	0.74 (0.02715)	0.6438 (0.0097)
L95% (cm)	187 (31)	316 (36)	215 (31)	202 (4)
% Fa/L (%cm^−1^)	1.453006 (0.1726)	0.8895 (0.0714)	1.2771 (0.126)	1.2279 (0.014)
JW/L (mL/min.cm)	− 5 × 10^–4^ (0.00039)	0.0003 (0.00012)	0.0001 (0.00026)	0.0006 (0.00026)
ARL (cm)	− 167	− 136	− 195	− 22

Values between brackets represent standard error to the mean (SE) (n = 3).

**Fig. 3:**
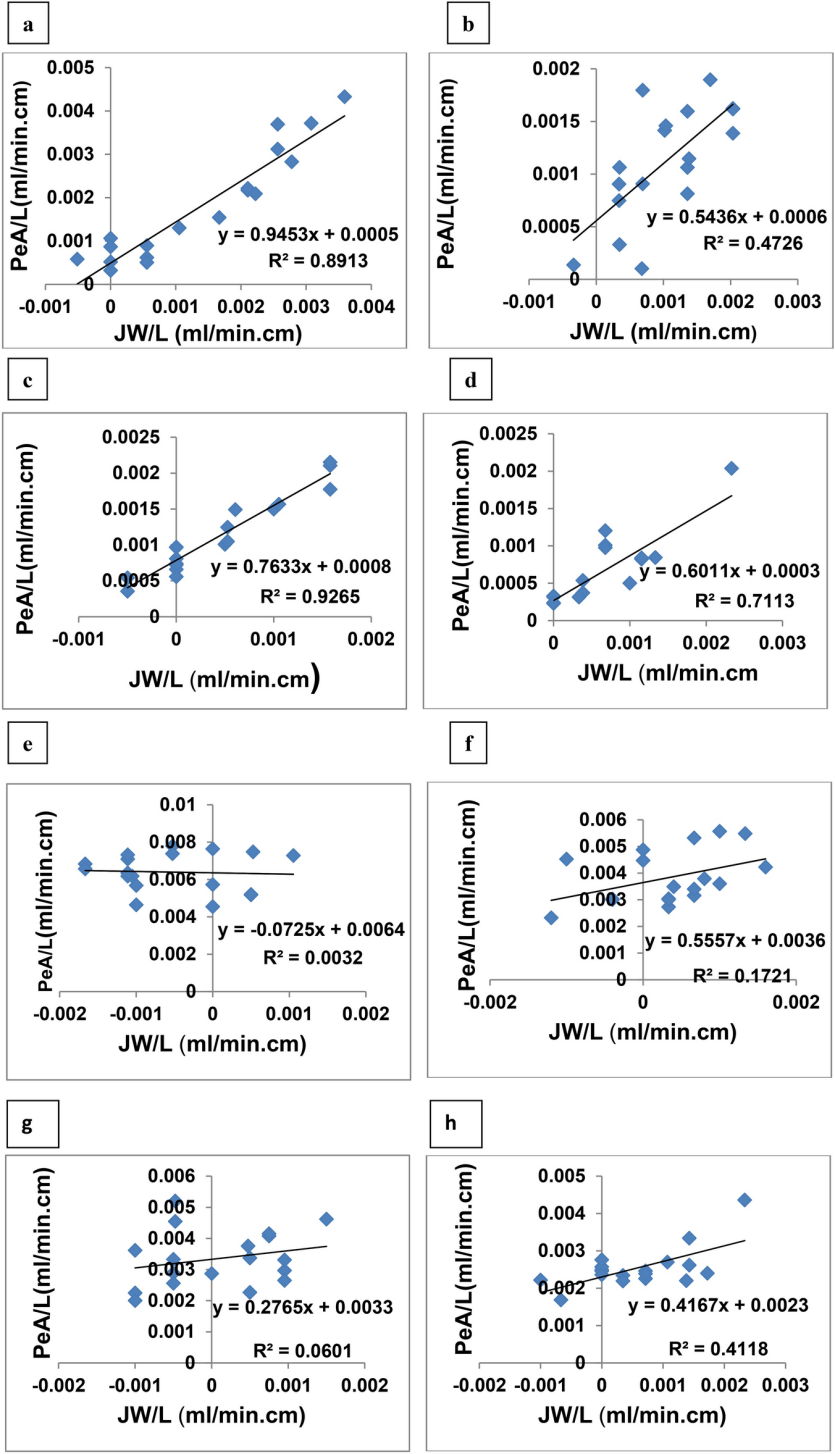
Absorptive clearance of hydrochlorothiazide from duodenum (left) and jejuno-ileum (right) as a function of the net water flux. The data obtained after perfusion of aqueous drug solution (**a**–**b**), fixed dose combination with lisinopril (**c**–**d**), span free glyceroniosome (**e**–**f**) and span containing glyceroniosome (**g**–**h**).

Delivering hydrochlorothiazide as aqueous solution resulted in incomplete absorption through the tested intestinal segments. This was clear from length required for almost complete absorption (L95%) which was 784 cm for duodenum and 1426 cm for jejuno-ileum. This was additionally indicated as negative ARL which were computed as − 764 and − 1246 cm in cases of duodenum and jejuno-ileum, respectively (Table 3). Figure 3 shows the effect of water flux and absorptive clearance of hydrochlorothiazide. This figure was used to compute the influence of transcellular and paracellular pathway in absorption of hydrochlorothiazide. This calculation reflected dominance of paracellular pathway in case of duodenum with 72.2% permeating through paracellular route. For jejuno-ileum 51.8% was due to paracellular route (Table 5). The increase in the contribution of transcellular pathway in case of jejuno-ileum compared with the duodenum can be due to the relative increase in surface area in jejuno-ileum segment. The overall contribution of paracellular absorption may be attributed to the increased water flux due to the diuretic effect of hydrochlorothiazide. The recorded incomplete absorption and the absorption pathways correlate with the published data on hydrochlorothiazide and confirm its categorization as class IV drug with poor solubility and permeability^10,41^.

**Table 5:** The percentage of drug Absorbed via transcellular and paracellular pathways from its aqueous solution or glyceroniosomal dispersion.

Aqueous Solution				
	Hydrochlorothiazide		Lisinopril	
Absorption Pathway	Duodenum	Jejuno-ileum	Duodenum	Jejuno-ileum
Transcellular (%)	27.8	48.2	53.3	32.1
Paracellular (%)	72.2	51.8	46.7	67.8

Fixed dose combination				
	Hydrochlorothiazide		Lisinopril	
Absorption Pathway	Duodenum	Jejuno-ileum	Duodenum	Jejuno-ileum
Transcellular (%)	71.2	41.2	76.4	12.1
Paracellular (%)	28.8	58.8	23.6	87.9

Span free glyceroniosome				
	Hydrochlorothiazide		Lisinopril	
Absorption Pathway	Duodenum	Jejuno-ileum	Duodenum	Jejuno-ileum
Transcellular (%)	100	93.9	95	93.2
Paracellular (%)	0	6.1	5	6.8

Span containing glyceroniosome				
	Hydrochlorothiazide		Lisinopril	
Absorption Pathway	Duodenum	Jejuno-ileum	Duodenum	Jejuno-ileum
Transcellular (%)	98.3	90.4	98.6	94.4
Paracellular (%)	1.7	9.6	1.4	5.6

For lisinopril, the ARL were calculated to be − 1472 and -600 cm in cases of the duodenum, jejuno-ileum, respectively. The calculated L95% values were 1492 cm for duodenum and 780 cm for jejuno- ileum (Table 4). With respect to the absorption pathways of lisinopril, the percentage paracellular absorption was 46.7% for the duodenum and 67.8% for jejuno-ileum (Table 5). The recorded incomplete absorption of lisinopril was shown in other studies with the absorption being minimized with increasing the concentration of drug perfused. This pattern was attributed to the possible contribution of carrier mediated transport which is a saturable process. The dominance of paracellular absorption was shown at higher concentration of lisinopril. The poor permeation was shown to be due to P-gp intestinal efflux^42^. It is important to highlight that the absorptive clearance per unit length (PeA/L) was higer in case of jejuno-ileum compared with the duodenum (Table 4). This may correlate with the site specific nature of carrier mediated transport which is more abundant in the jejunum^43^.

Co-perfusion of hydrochlorothiazide with lisinopril in the same aqueous solution resulted in non-significant change in the absorption parameters from the duodenum segment (p > 0.05) for each drug while in case of absorption from jejuno-ileum, the absorption parameters reduced significantly in case of lisinopril (p < 0.05) compared to the corresponding lisinopril solution which was pumped in absence of hydrochlorothiazide. This was reflected from significant decrease in the absorptive clearance per unit length (PeA/L) and the increase in the L95% values (Tables 3 and 4). The absence of significant effect in the duodenum with significant reduction in the permeation from jejuno-ileum suggests a possible effect for hydrochlorothiazide on the major absorption mechanism of lisinopril. Accordingly, it can be hypothesized that hydrochlorothiazide may interfere with the carrier mediated transport of lisinopril probably via competition. In absence of supporting literature and clear structure similarity, the ability of thiazide to hate peptide transporter requires verification. Hydrochlorothiazide was shown to be substrate for both organic anion and organic cation transporters in the kidney. This can open the way for possible transporter mediated absorption in the intestine which needs confirmation^44^.

Incorporation of hydrochlorothiazide into glyceroniosomes increased the intestinal absorption of the drug compared with the corresponding aqueous solution either alone or with lisinopril. The PeA/L was increased by 3.5 and 3.4-fold after perfusion of hydrochlorothiazide span free glyceroniosomes in case of duodenum, jejuno-ileum, respectively. Span containing glyceroniosomes were less effective recording 1.8 and 1.3-fold increase in PeA/L after perfusion through duodenum, jejuno-ileum, respectively. The increase in the absorptive clearance was reflected as an increase in the %Fa/L with significant reduction in value of L95% compared with the parameters recorded after perfusion of aqueous hydrochlorothiazide solution (P < 0.05, Table 3). Interestingly, the increase in the absorptive clearance was associated with noticeable increase in the contribution of the transcellular pathway in drug absorption from both segments which was shown from the plots of PeA/L as a function of water flux (Fig. 3). For example, the % transcellular absorption was increased from 27.8 to 100% and from 48.1 to 93.8% in case of duodemum and jejuno-ileum respectively after perfusion of span free glyceroniosomes (Table 5). In case of span containing glyceroniosomes, the % transcellular absorption was 98.2% in case of duodemum and 90.3% in jejuno-ileum. Niosomal systems were employed formerly to enhance oral bioavailability of lipophilic drugs^45,46^.

**Fig. 4:**
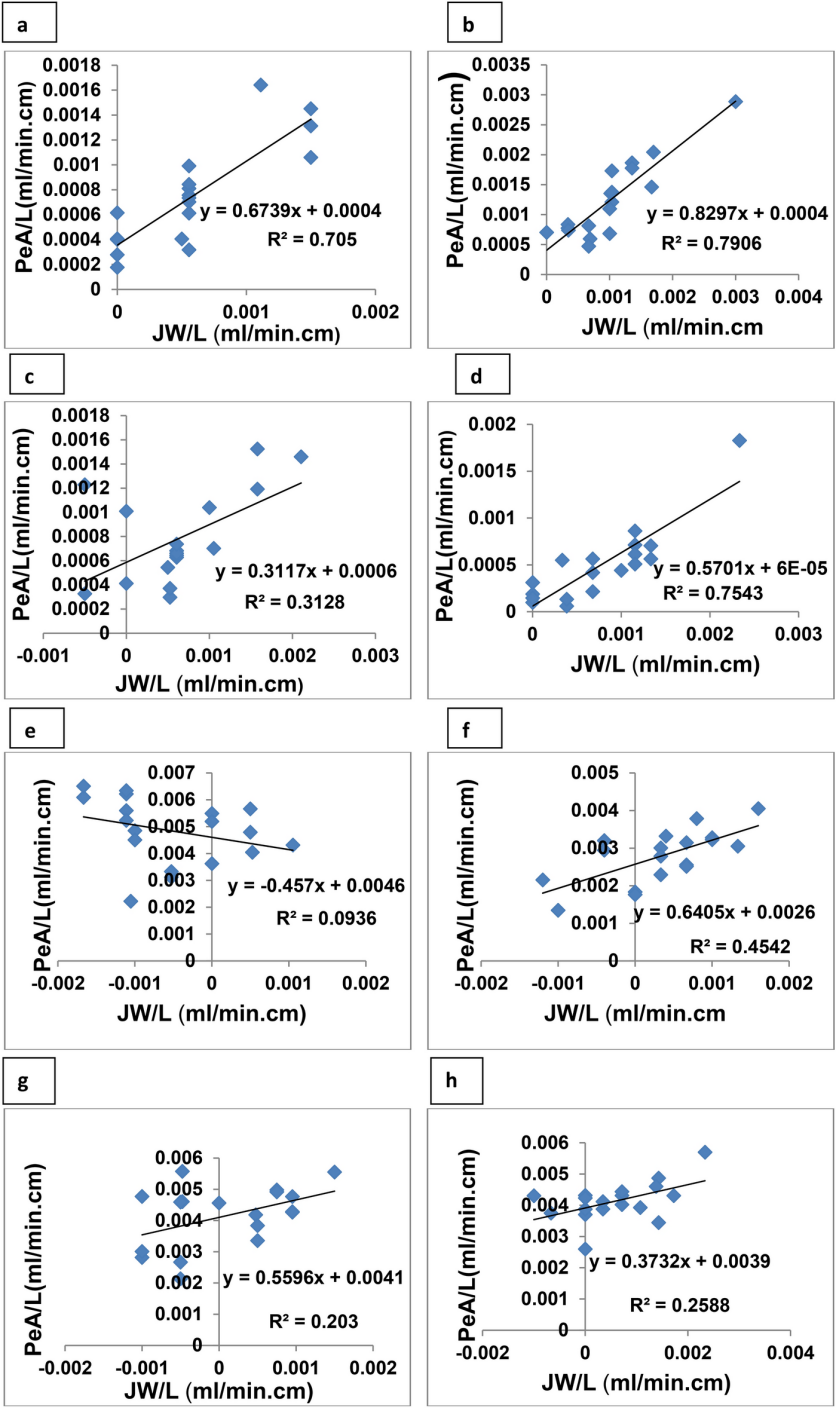
Absorptive clearance of lisinopril from duodenum (left) and jejuno-ileum (right) as a function of the net water flux. The data obtained after perfusion of aqueous drug solution (**a**–**b**), fixed dose combination with hydrochlorothiazide (**c**–**d**), span free glyceroniosome (**e**–**f**) and span containing glyceroniosome (**g**–**h**).

Perfusion of lisinopril glyceroniosomal dispersion improved the membrane transport parameters of the drug compared with simple aqueous solution in both duodenal and jejuno-ileum segments (Table 4). The PeA/L and %Fa of lisinopril were increased and L95% was shortened after drug encapsulation into glyceroniosomes (Table 4). This was tracked as a trend in the duodenum with the enhancement being significant in the jejuno-ileum segment (P < 0.05). Vesicular systems were successfully adopted to enhance oral absorption of hydrophilic drugs like lisinopril^47,48^. The ability of niosomes to deliver both ionized and unionized forms of the drug through intestinal membrane was also reported highlighting the feasibility of vesicular carrier for delivery of the hydrophilic species^14^. Considering the transport pathways of lisinopril after glyceroniosomal incorporation, the transcellular contribution to the drug absorption was amplified both in the duodenum and jejuno-ileum, indicating a preferential absorption pathway for such vesicular system (Table 5 and Fig. 4). The recorded increase in transcellular absorption dictates the possible mechanism of enhanced intestinal absorption supporting possible membrane fluidization and/or direct vesicle absorption.

Alternative mechanisms were suggested for niosomes as intestinal absorption enhancers. Like other lipid nanocarrier, they can exhibit membrane fluidizing effect increasing intestinal permeability. This will be expressed in transcellualr absorption fitting with our findings. This effect depends on the composition with formulation containing Tween 40 being more effective due to its fluid nature allowing greater ability to interact with biological membrane compared with Span 60. This explains the superiority of Tween containing formulation. Another supposition depends on the ability of lipid digestion product to widen the tight junctions providing greater chance for paracellular transport^49^. The later hypothesis did not operate for the current study due to lack of increase in the paracellular transport after vesicular delivery. Enhanced absorption via lymphatic transport was also considered as possible mechanism for enhanced intestinal absorption from lipid based systems and from vesicular systems like niosomses^14,50^. The later can offer the advantage of escaping the presystemic drug disposition^51^.

Incorporation of glycerol in vesicular system was employed to modify the properties of phospholipid-based vesicles (liposomes). The idea was to develop more fluid vesicles which will have greater ability to invade biological membrane. These vesicles were defined as glycerosomes and were able to improve skin deposition and permeation of diclofenac sodium. The results were explained on the base that glycerol hastened the ability of vesicular membrane to permeate through biological membrane^22^. Later on, glycerosomes were shown to enhance curcumin accumulation in the lungs after inhalation^52^. Glycerosomes were successfully applied as nasal delivery systems for lacidipine. This was based on ex-vivo investigation which resulted in 3.65-fold increase in drug permeation through membrane compared to lacidipine suspension^23^. Enhanced ex-vivo permeation was also shown through cornea^53^. These positive results can be extrapolated to intestinal membrane. Glycerosomes were regarded as a promising carrier to enhance the oral bioavailability and brain delivery of Quetiapine fumarate as it showed brain and plasma bioavailability enhancement compared to the drug suspension^54^. These investigations support our findings as the action of glycerol on liposomes can be extrapolated to niosomes and the action of glyceroniosomes can be similarly explained.

## Conclusion

Hydrochlorothiazide and lisinopril underwent incomplete absorption from duodenum and jejuno-ileum. Simultaneous intestinal perfusion of both drugs reduced the intestinal absorption of lisinopril. Glyceroniosomes were successfully developed for simultaneous encapsulation of hydrochlorothiazide and lisinopril. Glyceroniosomes were able to hasten the intestinal absorption of hydrochlorothiazide and lisinopril. Tween 40 containing glyceroniosomes were more efficient compared with Span 60 based vesicles.

## Data Availability

The datasets generated and/or analyzed during the current study are available from the corresponding author on reasonable request.
